# Supplementary figures and images for: Removal of cyanobacteria from a water supply reservoir by sedimentation using flocculants and suspended solids as ballast: Case of Legedadi Reservoir (Ethiopia)
Source: PLoS One. 2021 Apr 12;16(4):e0249720. doi: 10.1371/journal.pone.0249720 (PMC8041171; doi:10.1371/journal.pone.0249720)

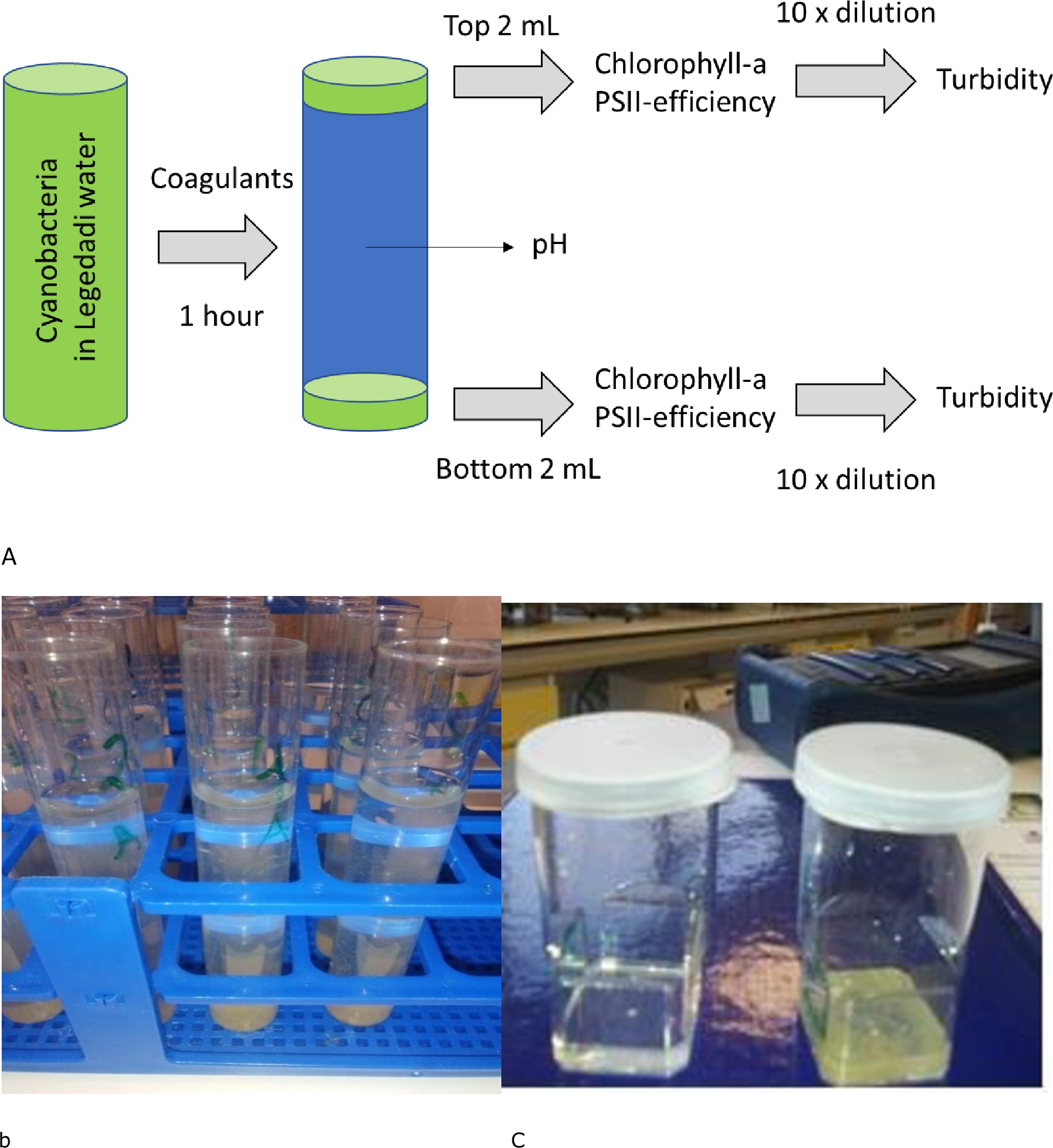

Supplement: S1 Fig — Experimental set-up of the Floc and Sink technique: (A) conceptual diagram, (B) Coagulation experiment with different coagulants (C) Top and bottom 2mL samples after 1hr experiment. (TIF) [file pone.0249720.s001.tif]
