# Supplementary material for: Removal of cyanobacteria from a water supply reservoir by sedimentation using flocculants and suspended solids as ballast: Case of Legedadi Reservoir (Ethiopia)
Source: PLoS One. 2021 Apr 12;16(4):e0249720. doi: 10.1371/journal.pone.0249720 (PMC8041171; doi:10.1371/journal.pone.0249720)
Supplement: S1 Table — (DOCX) [file pone.0249720.s002.docx]

Results of one-way ANOVA’s. Similar letters (A,...,E) per column indicate homogenoeus groups that are not different at the 95% level (Tukey’s test). For Photosystem II efficiencies (Ф_PSII_) noromality test failed and Kruskal-Wallis One Way Analysis of Variance on Ranks was run instead.

|  | CHL - top | CHL-bottom | Ф_PSII_ -top | Ф_PSII_ -bottom | pH |
| --- | --- | --- | --- | --- | --- |
| Control | A | A | A | A | A |
| Moringa | B | B | A | A | B |
| Moringa+Chitosan | B | C | A | A | C |
| PAC | A | D | A | A | D |
| Chitosan | C | A | A | A | E |
| *F-value: F_4,14_ =* | 179.859 | 322.995 | H_4_ = 11.249 | H_4_ = 8.841 | 66.329 |
| *P-value* | <0.001 | <0.001 | 0.024 | 0.065 | <0.001 |
| Normality Test (Shapiro-Wilk): | 0.252 | 0.409 | < 0.050 | < 0.050 | 0.943 |
| Equal Variance Test (Brown-Forsythe): | 0.260 | 0.535 | n.a. | n.a. | 0.089 |
