# Supplementary material for: Removal of cyanobacteria from a water supply reservoir by sedimentation using flocculants and suspended solids as ballast: Case of Legedadi Reservoir (Ethiopia)
Source: PLoS One. 2021 Apr 12;16(4):e0249720. doi: 10.1371/journal.pone.0249720 (PMC8041171; doi:10.1371/journal.pone.0249720)
Supplement: S1 File — (DOCX) [file pone.0249720.s003.docx]

**Descriptive Statistics:**

**Data source:** Data 1 in growth statistics

**Column Size Missing Mean Std Dev Std. Error C.I. of Mean**

TRW CU 30 0 876.256 1227.244 224.063 458.260

TRW AN 30 0 1055.401 2004.028 365.884 748.316

TRW MC 30 0 846.435 1447.218 264.225 540.400

WC CU 30 0 336.463 312.804 57.110 116.803

WC AN 30 0 647.052 1163.788 212.478 434.565

WC MC 30 0 593.175 776.653 141.797 290.007

RRW CU 30 0 369.239 231.265 42.223 86.356

RRW AN 30 0 397.607 261.896 47.815 97.793

RRW MC 30 0 472.454 384.188 70.143 143.458

CONTROL 30 0 1754.451 1776.958 324.427 663.527

**Column Range Max Min Median 25% 75%**

TRW CU 4044.750 4049.380 4.630 61.400 19.460 1888.885

TRW AN 8433.170 8437.320 4.150 57.820 20.000 1336.153

TRW MC 5303.140 5307.120 3.980 35.570 18.038 1218.253

WC CU 930.560 950.560 20.000 306.450 37.633 554.400

WC AN 3913.880 3913.880 0.000 46.590 11.870 696.700

WC MC 2280.810 2280.810 0.000 111.450 25.572 1446.735

RRW CU 1119.370 1135.190 15.820 389.290 175.855 494.365

RRW AN 1215.970 1268.820 52.850 382.300 170.005 557.127

RRW MC 1468.400 1489.800 21.400 342.635 162.318 726.530

CONTROL 5403.110 5515.090 111.980 1217.940 160.000 3246.810

**Column Skewness Kurtosis K-S Dist. K-S Prob. SWilk W SWilk Prob**

TRW CU 1.274 0.538 0.302 <0.001 0.742 <0.001

TRW AN 2.460 6.170 0.329 <0.001 0.602 <0.001

TRW MC 1.757 2.240 0.358 <0.001 0.650 <0.001

WC CU 0.618 -0.956 0.211 0.001 0.863 0.001

WC AN 2.285 4.266 0.289 <0.001 0.591 <0.001

WC MC 0.962 -0.805 0.302 <0.001 0.734 <0.001

RRW CU 0.954 2.868 0.135 0.169 0.914 0.018

RRW AN 1.296 2.823 0.104 0.516 0.908 0.014

RRW MC 1.226 1.185 0.191 0.007 0.878 0.003

CONTROL 0.846 -0.293 0.215 0.001 0.829 <0.001

**Column Sum Sum of Squares**

TRW CU 26287.680 66712451.480

TRW AN 31662.030 149883836.785

TRW MC 25393.060 82232346.902

WC CU 10093.900 6233778.990

WC AN 19411.560 51837935.511

WC MC 17795.240 28048183.909

RRW CU 11077.180 5641150.468

RRW AN 11928.200 6731822.299

RRW MC 14173.620 10976786.325

CONTROL 52633.530 183912765.192
